# Pancreatic Tail Cyst Causing Hydronephrosis

**DOI:** 10.7759/cureus.70534

**Published:** 2024-09-30

**Authors:** Andrej M Sodoma, Nicholas Bulba, James R Pellegrini, Jaspreet Singh, Antonio Costantino

**Affiliations:** 1 Internal Medicine, South Shore University Hospital, Bay Shore, USA; 2 Gastroenterology, New York Institute of Technology College of Osteopathic Medicine, Old Westbury, USA; 3 Internal Medicine, Nassau University Medical Center, East Meadow, USA; 4 Gastroenterology, South Shore University Hospital, Bay Shore, USA

**Keywords:** endoscopic ultrasound (eus), esophagogastroduodenoscopy (egd), obstructing cyst, obstructive hydronephrosis, pancreatic gastrostomy, pancreatitis, pseudocyst

## Abstract

Pancreatic cysts are abnormal masses found in the pancreas. They are either cancerous or benign and are mostly found incidentally on imaging. The majority are asymptomatic, but these cysts can sometimes become so large that they obstruct the function of structures around them. In this case, a pancreatic pseudocyst became so large that it caused hydronephrosis. An elderly female presented to a community hospital with abdominal pain. A computed tomography (CT) during that admission noted intraductal papillary mucinous neoplasm with ductal dilatation worrisome for underlying malignancy. An MRI was recommended but could not be performed due to incompatible hardware. The patient’s abdominal pain improved, so she was discharged for outpatient endoscopic ultrasound (EUS). Soon after discharge, the patient experienced worse right upper quadrant (RUQ) pain. A repeat CT upon her return to the hospital showed a new pancreatic pseudocyst causing left hydronephrosis. The patient was transferred to a tertiary care center for further management. An esophagogastroduodenoscopy (EGD) was performed, and an extensive peripancreatic fluid collection was identified, measuring approximately 11 cm × 7 cm. A 15 mm Hot AXIOS (LAMS) cystogastrostomy tube was placed, and the cytopathology report was negative for malignant cells. A repeat CT abdomen post-procedure showed near-complete resolution of pseudocyst. A repeat EGD was performed to monitor the cyst and remove the LAMS cystogastrostomy tube. The patient was discharged home with close follow-up with GI. Overall, pancreatic pseudocysts sometimes resolve, but if they do not, they require surgery. In this case, minimally invasive advanced endoscopy using a cystogastrostomy tube relieved the patient's obstruction.

## Introduction

Pancreatic cysts are cancerous, precancerous, or benign masses in the pancreas [1]. Cyst characteristics suggestive of malignancy are size, wall thickness, main pancreatic duct dilation, and mucin content [2]. Roughly 71% of these masses are found incidentally, as most people with them are asymptomatic [3]. The prevalence of pancreatic cysts is increasing due to improvements in imaging technologies [4]. Common imaging modalities are computed tomography (CT) and magnetic resonance cholangiopancreatography (MRCP), but the accuracy is around 68% [4]. Endoscopic ultrasound (EUS) has been increasingly applied to evaluate pancreatic cysts as the imaging modality has increased diagnosis accuracy and sampling capability [5]. 

Pancreatic pseudocysts can become so large that they compress nearby structures, including the biliary system, intestines, and stomach, causing symptoms such as abdominal pain, bloating, steatorrhea, light-colored stools, and dark urine. However, these symptoms do not always occur. In this case report, a pancreatic tail cyst grew so large that it caused abdominal pain and hydronephrosis. 

## Case presentation

**Figure 1:**
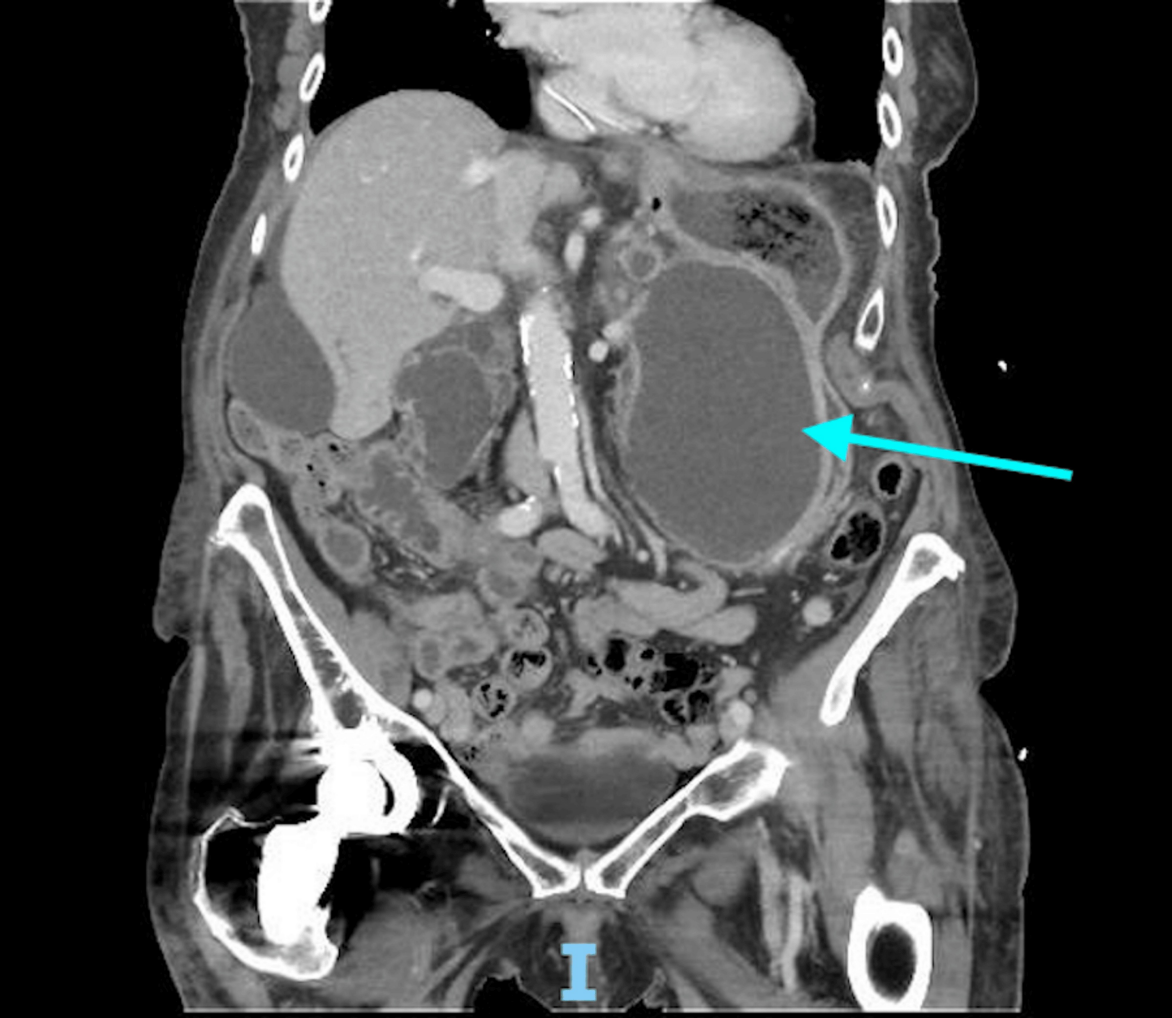
CT of the abdomen and pelvis with intravenous contrast performed on readmission to the tertiary care center. CT: computer tomography.

A 95-year-old woman was transferred to the emergency department of a tertiary care center from a local community hospital for recurrent acute pancreatitis. Initially, the patient was scheduled for an EUS after being discharged from the community hospital for acute pancreatitis a few days ago due to a concern for a malignant pancreatic mass. The CT showed an intraductal papillary mucinous neoplasm (IPMN) with ductal dilatation worrisome for underlying malignancy (Figure 1). The patient could not undergo an MRI due to pacemaker incompatibility with MRI. She had a past medical history of heart failure with preserved ejection fraction (HFpEF), sick sinus syndrome requiring a pacemaker, and chronic atrial fibrillation (AFib). 

**Figure 2:**
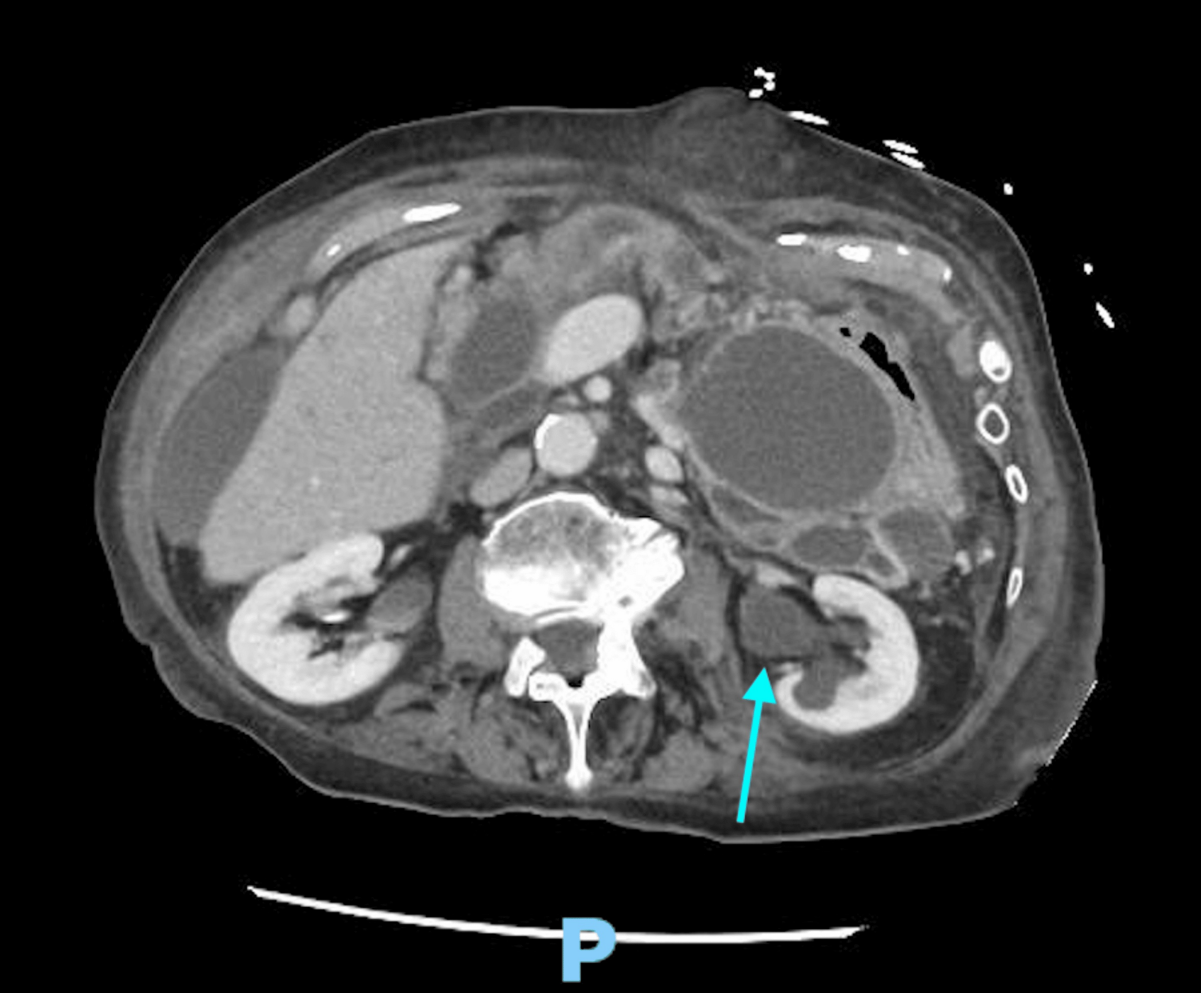
CT abdomen and pelvis with oral and intravenous contrast showing hydronephrosis.

On admission to the tertiary care center, the patient had normal vitals and no fever. Her labs on admission were leukocytes of 15.71 K/uL, no signs of anemia, Na of 127 mmol/L, Cl of 91 mmol/L, and lipase of 8 U/L, which never became more elevated, and liver function tests (AST/ALT/ALP) were typical throughout admission. HbA1c was 8.7%. A urinalysis was also performed, which showed numerous bacteria, moderate blood, 110 RBCs, and no leukocytes in urine. A CT scan of the abdomen with contrast revealed a new pancreatic pseudocyst causing left hydronephrosis, which was absent on the prior exam, and no renal stones (Figure 2). Carcinoembryonic antigen (CEA) was taken, which was elevated at 19.5 ng/ml. CA 19-9 was also elevated at 64 U/ml. 

**Figure 3:**
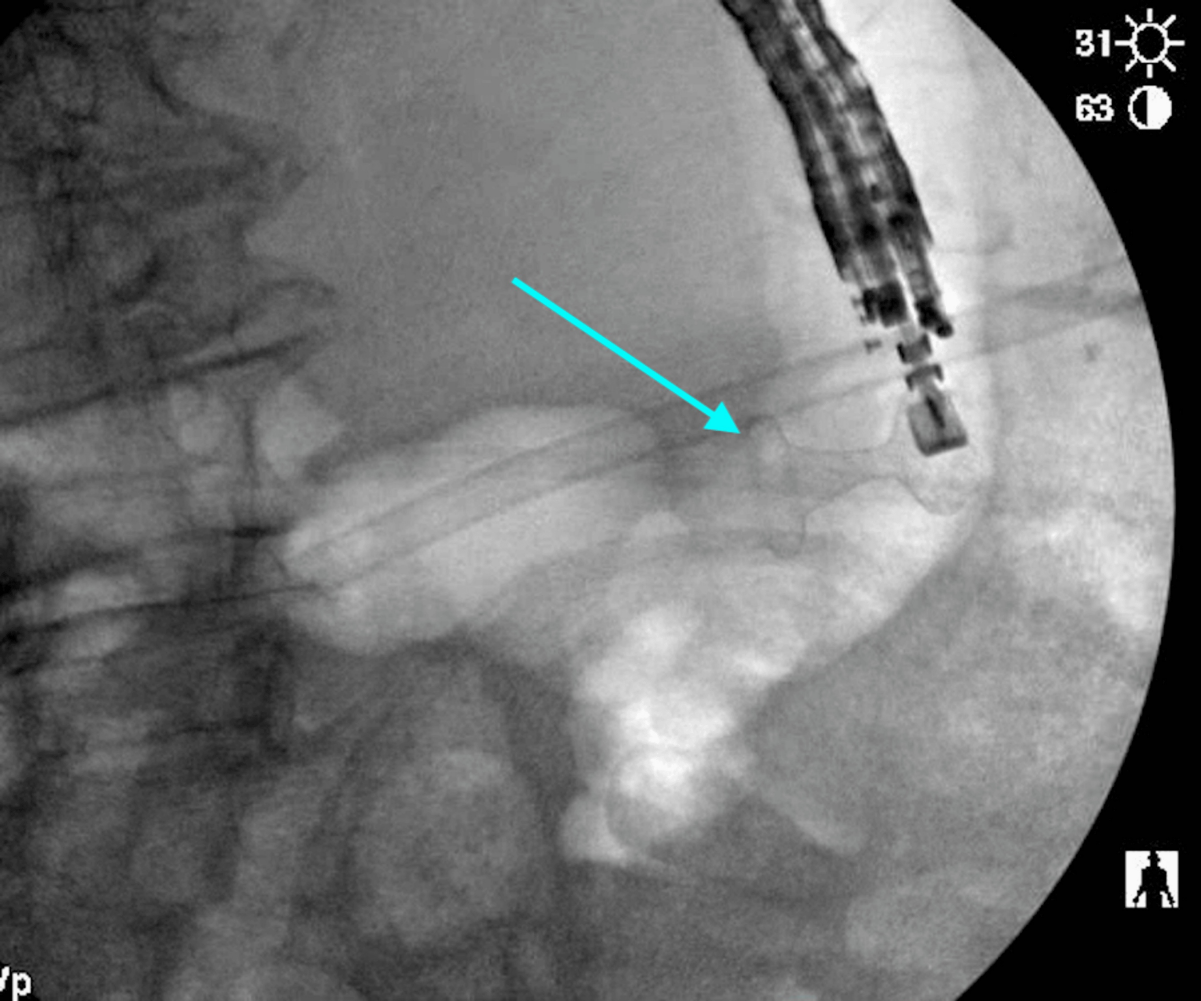
Initial EGD showing the placement of a gastrostomy tube into the stomach to the pancreatic tail cyst. EGD: esophagogastroduodenoscopy.

The patient then underwent esophagogastroduodenoscopy (EGD) and EUS with cyst gastrostomy drainage. An extensive peripancreatic fluid collection, measuring approximately 11 cm × 7 cm, was identified. A 15 mm Hot AXIOS (lumen-apposing metal stent (LAMS)) cystogastrostomy tube (Boston Scientific Corporation, Marlborough, MA, USA) was placed. The pancreatic fluid and a fine-needle biopsy were sent for a cytopathology report, which was negative for malignant cells (Figure 3).

**Figure 4:**
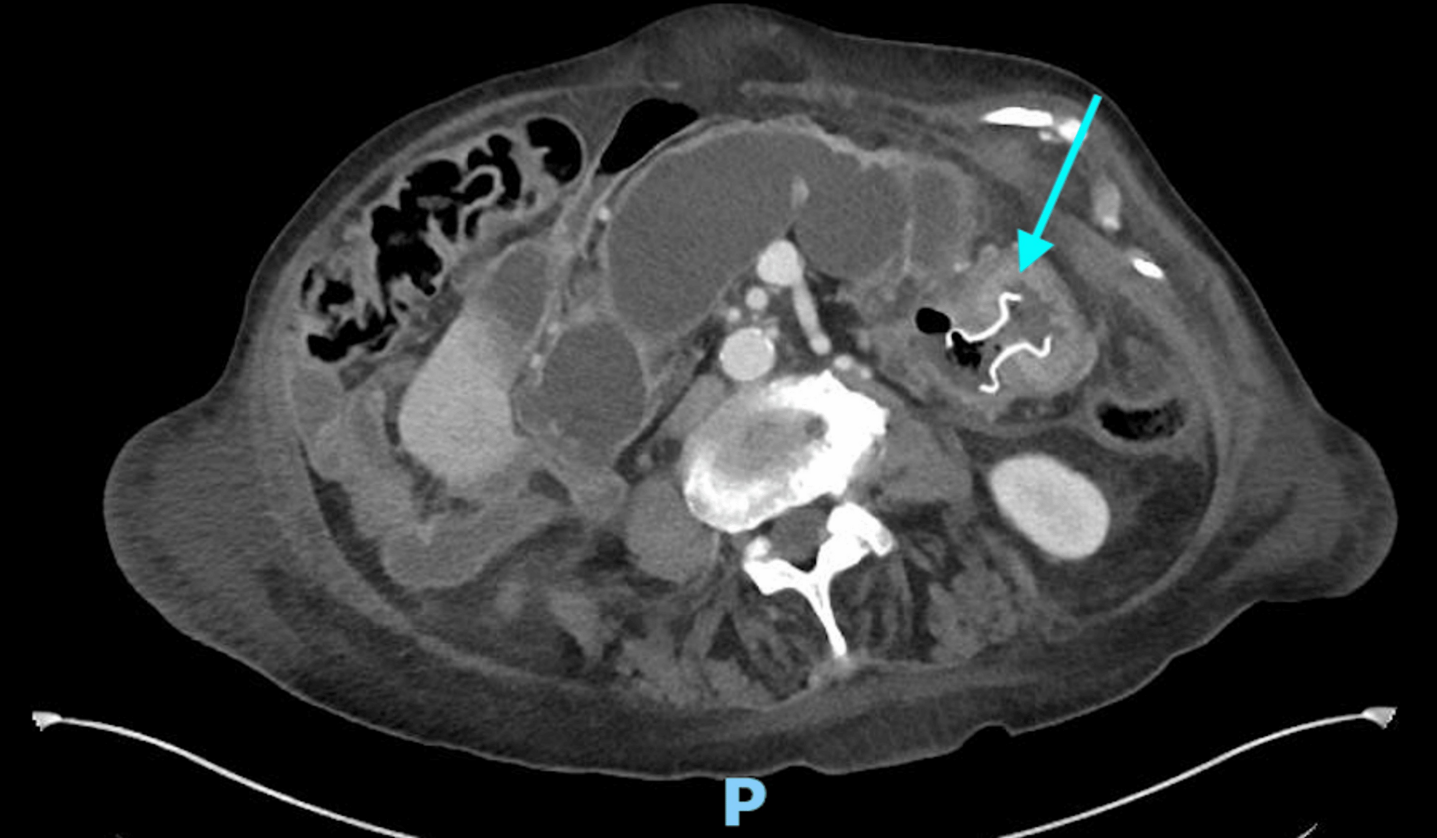
CT of the abdomen and pelvis with intravenous contrast after the placement of the AXIOS stent. CT: computer tomography.

Repeat CT abdomen post-procedure showed the AXIOS drain in place, with near-complete resolution of pseudocyst at the pancreatic tail and stable severe dilatation of the pancreatic duct (Figure 4).

A repeat EGD was performed, showing the full resolution of the prior pseudocyst. The cystogastrostomy LAMS tube was removed and confirmed to be intact. The patient tolerated a diet shortly after the procedure, with no complaints, abdominal tenderness, afebrile, or leukocytosis. The patient was discharged home with a close follow-up with the gastrointestinal (GI) specialist. 

## Discussion

Pancreatic pseudocysts are collections of necrosis walled off by granulation tissue and fibrosis stemming from acute pancreatitis, chronic pancreatitis, trauma, biliary stones, or idiopathic origins [6]. As many as 70-78% of cases are acute or chronic; most commonly, they occur due to increased alcohol use [7]. These benign growths account for two-thirds of all pancreatic cyst lesions and have presenting symptoms such as abdominal pain, nausea, vomiting, jaundice, and fever [7]. 

Intraductal papillary mucinous neoplasms (IPMNs) are tumors that develop from the pancreatic ducts and produce mucin [8]. The majority are benign but can progress to cancer [8]. Many differences exist between pseudocysts and IPMNs. First is the presentation of the history of pancreatic pseudocysts, which tend to occur in patients with acute or chronic pancreatitis; IPMNs usually have no history. Second, radiographic findings of IPMNs show a patulous, mucin-extruding papillary orifice when the IPMN is in the main duct [9]. Third is pseudocyst fluid analysis with low CEA, high lipase, and amylase, while IPMNs showed high CEA [9]. 

In this case, the patient presented to the hospital with abdominal pain that was diagnosed with CT; the results resembled IPMN with ductal dilatation. The patient’s symptoms initially improved. However, upon readmission to the hospital, the patient complained of right upper quadrant (RUQ) pain, nausea, and decreased oral intake. The history correlated more with a pseudocyst due to a history of abdominal pain. The CT scan confirmed a new pancreatic pseudocyst and secondary left hydronephrosis. However, the lipase was not elevated, but the CEA was. 

Although complications of pseudocysts can occur with varying degrees of severity ranging from infection and rupture, to compression of nearby structures, compression of the ureters leading to hydronephrosis is considered very rare [6]. Hydronephrosis is a complication of unilateral or bilateral dilation of the renal collecting ducts, including the renal pelvis, ureters, or calyces [10]. Many causes of this complication can originate from an intrinsic obstruction or extrinsic compression of the renal system [10]. Two leading causes of hydronephrosis secondary to a pseudocyst include compression of the kidney with subsequent blockage of the ureter and enzymatic degradation of the kidney structures due to pancreatic leakage [11]. One previous report found evidence of an ectopic pancreas pseudocyst in the kidney, causing secondary hydronephrosis [12]. Although this is very rare, the proximity of the cyst to the kidney made the presenting symptoms attributable [12]. In this case, the patient developed left hydronephrosis due to a cyst that originated from the pancreas, likely due to the adjacency of the pancreas to the left renal fascia. One study highlighted this link between fascial planes, reporting that the left kidney in patients tended to be affected more often than the right [13]. This study also performed a five-year review of 207 patients with acute pancreatitis who underwent a CT. This resulted in 3% of patients diagnosed with secondary hydronephrosis, a much higher-than-expected occurrence [6]. 

In this case, the pancreatic cyst was drained, and the fluid sent for cytology was found to be non-malignant. The prognosis of pancreatic cysts depends on their contents. Pancreatic pseudocysts sometimes resolve in two to six weeks; if they do not, they require surgery or endoscopy. Treating pancreatic pseudocysts remains controversial as conservative treatment is initiated since spontaneous resolution occurs after most acute or chronic pancreatitis cases [7]. If pseudocysts persist after six weeks or pose life-threatening circumstances, the three invasive treatments currently used are percutaneous, endoscopic, and surgical drainage (SD) [6]. Advancements in surgery have made cystogastrostomy and cystojejunostomy solutions with a great success rate. In this case, the patient underwent an EGD and EUS with cystogastrostomy drainage, which showed an extensive peripancreatic fluid collection. According to multiple reports, endoscopic drainage (ED) with ultrasound (US) guidance has had a higher success rate than performing ED alone [7]. It has been reported that utilizing ED with the US yields a shorter hospital stay and decreased patient costs compared to only SD, proving it to be the preferred current treatment and the treatment performed in this case [7]. This case resembled an ideal circumstance and usage of a SD treatment, showcasing the importance of early intervention to prevent pancreatic and renal complications.

## Conclusions

This case investigates a unique presentation of a pancreatic pseudocyst with unilateral left hydronephrosis. While pancreatitis leading to the formation of this cyst is not considered a rare occurrence, the involvement of the kidney and subsequent hydronephrosis raises concern for future cases and their treatment. The involvement of organs proximal to the pancreas has always been considered a complication, but now renal diagnoses should be on the differential. This case also presented a treatment plan for endoscopic drainage with EUS, proving successful in theory and practice. In the future, research can examine further conservative versus surgical treatments for cases like this. This patient performed well under cystogastrostomy, and further symptoms were resolved.
